# Characterization of the Inclusion Complexes of Isothiocyanates with γ-Cyclodextrin for Improvement of Antibacterial Activities against *Staphylococcus aureus*

**DOI:** 10.3390/foods11010060

**Published:** 2021-12-27

**Authors:** Jianan Liu, Hongyan Wu, Xinying Ao, Hongshun Hao, Jingran Bi, Hongman Hou, Gongliang Zhang

**Affiliations:** 1 School of Food Science and Technology, Dalian Polytechnic University, Dalian 116034, China; ljn1263778194@163.com (J.L.); wuhongyan1908@hotmail.com (H.W.); 13756004290@163.com (X.A.); bijingran1225@foxmail.com (J.B.); houhongman2011@hotmail.com (H.H.); 2 Department of Inorganic Nonmetallic Materials Engineering, Dalian Polytechnic University, Dalian 116034, China; beike1952@163.com; 3 Liaoning Key Lab for Aquatic Processing Quality and Safety, Dalian 116034, China

**Keywords:** cyclodextrin, inclusion, isothiocyanates, *Staphylococcus aureus*, controlled release, antibacterial activities

## Abstract

The aim of this study was to develop inclusions formed by γ-cyclodextrin (γ-CD) and three isothiocyanates (ITCs), including benzyl isothiocyanate (BITC), phenethyl isothiocyanate (PEITC), and 3-methylthiopropyl isothiocyanate (MTPITC) to improve their controlled release for the inhibition of *Staphylococcus aureus* (*S. aureus*). These inclusion complexes were characterized using X-ray diffraction, Fourier-transform infrared, thermogravimetry, and scanning electron microscopy (SEM), providing appropriate evidence to confirm the formation of inclusion complexes. Preliminary evaluation of the antimicrobial activity of the different inclusion complexes, carried out in vitro by agar diffusion, showed that such activity lasted 5–7 days longer in γ-CD-BITC, in comparison with γ-CD-PEITC and γ-CD-MTPITC. The biofilm formation was less in *S. aureus* treated with γ-CD-BITC than that of BITC by using crystal violet quantification assay and SEM. The expression of virulence genes, including *sarA*, *agr*, *cp5D*, *cp8F*, *clf*, *nuc*, and *spa*, showed sustained downregulation in *S. aureus* treated with γ-CD-BITC for 24 h by quantitative real-time polymerase chain reaction (qRT-PCR). Moreover, the growth of *S. aureus* in cooked chicken breast treated with γ-CD-BITC and BITC was predicted by the Gompertz model. The lag time of γ-CD-BITC was 1.3–2.4 times longer than that of BITC, and correlation coefficient (R^2^) of the secondary models was 0.94–0.99, respectively. These results suggest that BITC has a more durable antibacterial effect against *S. aureus* after encapsulation by γ-CD.

## 1. Introduction

*Staphylococcus aureus* (*S. aureus*) is one of the most frequent causes of food poisoning [1]. *S. aureus* has the ability to adhere to various surfaces and create thick biofilms [1,2]. A self-produced matrix of extracellular polymeric molecules encases these surface-attached aggregated microbial populations, which allows them to withstand a variety of stressors and thrive in hostile settings [3]. Biofilm production, in fact, is one of the most important qualities that contributes to the successful growth of these bacteria, and it is thought to be necessary for the onset of their pathogenicity and persistence [4]. In addition, this nonmotile, catalase, and coagulase-positive coccus has a wide range of virulence traits that allow it to persist in a living host [5]. Consumers may be exposed to *S. aureus* infection through the processing or production of dairy, cooked meat, eggs, aquatic products, beans, ready-to-eat meals, and fresh vegetables [6]. Therefore, certain steps are needed to prevent *S. aureus* infection in order to maintain food safety.

Natural chemical products have been reported to exhibit remarkable antibacterial activities [7]. Isothiocyanates (ITCs), which are found in the Cruciferae family of plants, have long been used as antibacterial agents [8,9]. The antibacterial activities of 3-methylthiopropyl isothiocyanate (MTPITC), phenethyl isothiocyanate (PEITC), and benzyl isothiocyanate (BITC) against *S. aureus* have been reported by Wilson and Kim et al. [10,11]. Both PEITC and BITC inhibit the activity of methicillin-resistant *S. aureus* [12]. In addition, Yang et al. also found that BITC and PEITC showed strong antibacterial activities against toxin-producing *Escherichia coli* (*E. coli*) and enterotoxigenic *E. coli*, respectively [13]. ITCs have strong volatility, which diminishes their antibacterial activities and hinders their potential use as effective bioactive agents [14,15]. The use of carrier systems to encapsulate ITCs, thus achieving controlled release, is important to improve the inhibitory effect of ITCs on pathogenic bacteria.

Encapsulation is a beneficial method for protecting flavors, improving their sustained release characteristics, increasing their stability, and extending their shelf life [16,17]. The incompatibility of ITCs with many food packaging substances limits their application as antimicrobials due to their appearance in the form of oils [18]. These issues have been overcome by cyclodextrins (CDs). CDs are cyclic oligosaccharides composed of (1,4)-glycosidic-bonded glucopyranose units. These torus-like macro rings have relatively hydrophobic internal cavities rather than hydrophilic external cavities [19]. The inclusion process that occurs in water involves the displacement of water molecules by polar molecules of guests from the relatively hydrophobic cavities of CDs and stabilizes the encapsulated guest molecules mainly through the formation of many van der Waals attractive forces [20]. Different chemical compounds classes were complexed into cyclodextrin with antibacterial activities, including d-limonene [21], *Hyptis martiusii Benth* essential oil [22], and α-bisabolol [23]. γ-Cyclodextrin (γ-CD) is suitable for complexation due to its wide cavity and water solubility [24]. Phunpee et al. used a spray drying approach to create inclusion complexes between citral and CDs, revealing that a larger cavity promoted better geometric accommodation of citral in the order of γ-CD > β-CD > α-CD [25]. γ-CDs with good loading and release properties are extremely desirable as carrier materials for inhibitory applications. Moradi et al. found that thyme oil inclusion complex with γ-CD showed good controlled release and antibacterial activity against *S. aureus* [26]. It was demonstrated that electrospun zein nanofibrous webs encapsulated in the thymol/γ-CD inclusion complex (zein-THY/γ-CD-IC-NF) had been shown to suppress the development of *S. aureus* more effectively than that of zein-THY-NF [27]. However, no studies have been conducted to prepare ITC inclusions using γ-CD as a coating material and to investigate their controlled release and antibacterial activities.

In this study, we attempted to prepare ITC inclusion complexes using the freeze-drying method with γ-CD as the raw material. The focus of the study was to improve the stability of ITCs and to increase their antibacterial activity. The inclusion complexes were characterized using Fourier-transform infrared (FTIR), thermogravimetry (TGA), X-ray diffraction (XRD), and scanning electron microscopy (SEM). In addition, the controlled release, antibiofilm activity, effect on virulence gene expression and antibacterial activity of ITCs and γ-CD-ITCs against *S. aureus* in cooked chicken breast were also investigated.

## 2. Materials and Methods

### 2.1. Chemicals and Bacterial Strains

**Figure 1:**
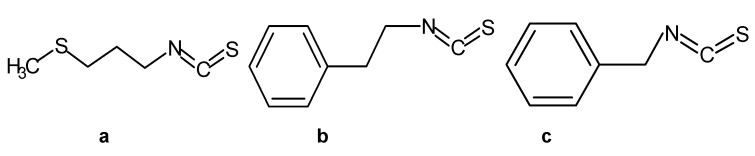
Structures of isothiocyanates (ITCs). (**a**) 3-methylthiopropyl isothiocyanate (MTPITC), (**b**) phenethyl isothiocyanate (PEITC), and (**c**) benzyl isothiocyanate (BITC).

γ-CD was purchased from Beijing baoxidi Technology Co., Ltd. (Beijing, China). ITCs, including MTPITC (purity > 99%), PEITC (purity > 99%), and BITC (purity > 99%), were purchased from Sigma, Burlington, MA, USA. Dimethyl sulfoxide was purchased from Damao (Tianjin, China). Glacial acetic acid was purchased from Aladdin (Shanghai, China). Their structures are shown in Figure 1. Chicken breast was purchased from the local market (Dalian, China). The gram-positive bacterium *S. aureus* ATCC 6538 was taken from the Dalian Polytechnic University Food Microbiology Laboratory (Dalian, China).

### 2.2. Preparation of the Inclusion Complexes

γ-CD was stirred in 100 mL of ultrapure water to obtain an aqueous solution at 15.41 mmol/L. The γ-CD solution was added with an ethanolic solution of 1.541 mmoL ITCs, and stirred magnetically at 60 °C for 3 h. The combined solution was then snap-frozen and freeze-dried for two days using Vacuum Freeze Dryer (GOLD SIM, Seattle, WA, USA).

### 2.3. Characterization of the Inclusion Complexes

γ-CD and the inclusion complexes (1 mg) were dissolved in ethanol at room temperature. The UV scanning spectra were obtained in the wavelength range of 200–350 nm by using a spectrophotometer (UV-2550, Shimadzu, Kyoto, Japan). Fourier transform infrared (FTIR) spectroscopy investigations of γ-CD-ITCs were performed in an FTIR Spectrum (PerkinElmer, Norwalk, CT, Japan), with an average of 64 scans per sample in 4000 to 500 cm^−1^. Thermogravimetric analysis (TGA) experiments were carried out using a thermogravimetric analyzer (TGA 550, TA, TA Instruments, New Castle, DE, USA) with a heating rate of 20 °C min^−1^ and a flow rate of 20 mL/min under nitrogen environment. A diffractometer (XRD-6100, Shimadzu, Kyoto, Japan) was used to measure X-ray diffraction (XRD) from 5 to 60° at 5° min^−1^ scanning speed with Cu Kα radiation (λ = 1.54060 Å). Moreover, the morphology analysis was measured by scanning electron microscopy (SEM) (Quanta 450, Waltham, MA, USA).

### 2.4. In Vitro Release Study

γ-CD-ITC inclusion complexes (100 mg) were dissolved in 20 mL of water and added with 20 mL of hexane. The sample solution was kept in a shaker at 37 °C for 48 h. The release amount of ITCs was spectrometrically assayed every 4 h at 247 nm. The volume was replaced with fresh hexane after each estimation. The experiments were carried out in triplicate.

### 2.5. Antibacterial Assays

MTPITC, PEITC, and BITC and the corresponding inclusion complexes (based on the BITC concentration) with the same final concentration at 0.25 mmol/L were added to the bacterial suspension and cultured at 37 °C in a shaker at 120 rpm. The absorbance at 600 nm was measured every 4 h. The growth curve was further obtained. In addition, the antibacterial activities of ITCs were also studied using the agar diffusion method referencing Goni et al. [28] with slight modifications. Briefly, the inhibitory effect of ITCs and γ-CD-ITCs on *S. aureus* was tested by punching a hole on the Luria–Bertani broth (LB) agar medium. On the plate, 100 mL of *S. aureus* suspension (1 × 10^7^ CFU/mL) was distributed. Then, 1μmoL of ITCs (BITC, PEITC, and MTPITC) and γ-CD-ITCs (γ-CD-BITC, γ-CD-PEITC, and γ-CD-MTPITC, ITCs = 1 μmoL) were added to the wells of the plate. To avoid nutrient loss from the agar, the nutrient-rich plates were incubated at constant humidity for 10 days with the bacterial solution on them. Their antibacterial activities were compared by measuring and photographing the diameter of the inhibition zone (DIZ). The MIC was determined by using the broth microdilution technique [29]. Different concentrations of BITC and *S. aureus* suspensions were added to sterile 96-well microplates and incubated for 12 h at 37 °C. The control was the Mueller–Hinton Broth with or without bacterial cultures.

### 2.6. Biofilm Analysis

#### 2.6.1. Crystal Violet Quantitative Assay

A suspension of about 10^7^ CFU/mL *S. aureus* was put to each well of a 96-well polystyrene plate, along with BITC at concentrations of 0, 1/8MIC, 1/4MIC, 1/2MIC, and γ-CD-BITC (based on the BITC concentration), and the plate was incubated at 37 °C for 48 h. After the biofilm was formed, the planktonic cells were removed using sterile PBS, and the biofilm was fixed with methanol for 15 min. The preserved biofilms were dyed with 1% crystal violet solution for 10 min. Finally, 33% glacial acetic acid (200 μL) was added to each well, and absorbance was read at 590 nm using a microplate reader (SpectraMax M2, Molecular Devices, Madison, WI, USA).

#### 2.6.2. SEM Analysis

*S. aureus* suspension (about 10^7^ CFU/mL) was prepared and incubated for 48 h at 37 °C with BITC and γ-CD-BITC (basing on BITC concentration) at concentrations of 0, 1/8MIC, 1/4MIC, and 1/2MIC on a coverslip (4 mm × 4 mm) in a 6-well polystyrene plate. Following the formation of the biofilm, the samples were collected and processed as described in our earlier study [30]. The gold-coated dehydrated *S. aureus* biofilm samples were examined under an SEM (Quanta 450, Waltham, MA, USA).

### 2.7. Quantitative Real-Time Polymerase Chain Reaction (qRT-PCR)

*S. aureus* (about 10^7^ CFU/mL) was treated with 1/4MIC BITC and γ-CD-BITC (based on the BITC concentration) for 24 h. The total RNA was extracted using the RNAprep Pure Cell/Bacterial Kit (Tiangen Biotech, Beijing, China). The total RNA was processed with the Prime ScriptTM RT kit from the gDNA Eraser (TaKaRa, Dalian, China) to remove genomic DNA and use reverse transcription to validate differential gene expression. qRT-PCR was performed on SYBR ^®^ Premix Ex Taq ^™^ II (TliRNaseH Plus) (Takara, Dalian, China). Seven virulence genes, including accessory gene regulator (*agr*), accessory gene regulator protein A (*sarA*), capsular polysaccharide biosynthesis protein (*cp5D*), capsular polysaccharide synthesis enzyme (*cp8F*), thermonuclease (*nuc*), clumping factor (*clf*), and protein A (*spa*) in the *S. aureus* strain ATCC 6538 were studied. The 16S rRNA gene was used as the endogenous gene, and the 2^−∆∆Ct^ method was applied to assess differential gene expression levels [31]. Table S1 lists the specific primers designed by Primer 5.0 software.

### 2.8. Primary Modeling

Fresh chicken breast was cut into cube blocks (approximately 25 g). The sterile cooked chicken breast samples were then obtained by sterilizing the cube blocks for 20 min at 121 °C. Samples of the BITC (or γ-CD-BITC) at 0.25 mmol/L and the bacterial suspension (about 10^7^ CFU/mL) were added, followed by homogenization for 120 s in a stomacher (400 Circulator, Seward, England). The inoculated samples were stored by using aseptic bags at 10, 15, 20, and 25 °C, respectively. At each sampling time point, three chicken samples were tested for microbiological examination.

The data of *S. aureus* in the cooked chicken breast treated with BITC and γ-CD-BITC (basing on BITC concentration) were modeled using the modified Gompertz model [6,32] as followed:

(1) $$Y\left(t\right)=y_{0}+\left(y_{max}-y_{0}\right)\times \exp\left\{-\exp\left[\frac{\mu_{max}e}{y_{max}-y_{0}}\left(\lambda -t\right)+1\right]\right\}$$

where *y*_0_, *y*_max_, and *Y*(*t*) are the bacterial population counts in a natural logarithm of *S. aureus* at initial, maximum, and time (Log CFU/g); *μ_max_* is the maximum specific growth rate (Log CFU/g/h); and *λ* is the lag time duration (h).

### 2.9. Secondary Modeling

Temperature, maximum specific growth rate (SGR), and lag time (LT) were used in secondary modeling with the polynomial model equation. The secondary models of SGR and LT were created using the polynomial model equation. The equations are the following:

(2) $$\mathrm{Polynomial}\;\mathrm{model}\;\mathrm{equation}=a+b\times T+c\times T^{2}$$

where, a, b, and c are constants and *T* is storage temperature.

### 2.10. Validation of Predictive Models

The bias factor (*Bf*), accuracy factor (*Af*), and correlation coefficient (*R*^2^) were utilized in this study to evaluate the performance of the projected models [33]. The equations for *Bf*, *Af*, and *R*^2^ are the following:

(3) $$\begin{array}{c} Bf=10^{\frac{\sum^{}\log\left(\frac{pred}{obs}\right)}{n}}\\ Af=10^{\frac{\sum^{}\left|\log\left(\frac{pred}{obs}\right)\right|}{n}}\\ R^{2}=1-\frac{\sum_{i=1}^{n}{\left(y_{i}-y_{i}\prime \right)}^{2}}{\sum_{i=1}^{n}{\left(y_{i}-\bar{y}\right)}^{2}} \end{array}$$

where *y*_*i*_ is the predicted value of the *i*th sample; *y*_*i*_′ is the measured value of the *i*th sample; and *n* is the number of samples.

### 2.11. Statistical Analysis

Statistical analysis of data (at least from three separate experiments) was done using one-way ANOVA analysis with Duncan test for comparison of individual means by SPSS software (IBM Inc., Armonk, NY, USA). In all cases, the significance level was set at *p* < 0.05 on *p* < 0.01. GraphPad Prism 8.0.2 was used to perform calculations of the parameter-adjusted Gompertz model.

## 3. Results and Discussion

### 3.1. Characterization of the ITCs Inclusion Complexes with γ-CD

**Figure 2:**
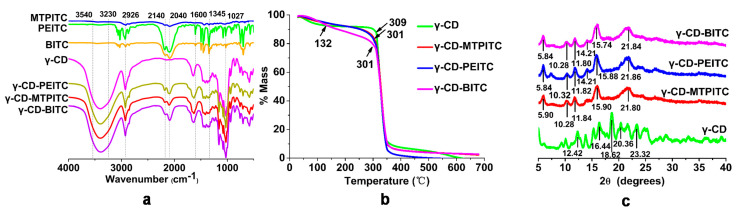
(**a**) FTIR spectra of ITC, γ-CD and γ-CD-ITCs. (**b**) TGA curves for γ-CD and γ-CD-ITCs. (**c**) X-ray diffraction patterns of γ-CD and γ-CD-ITCs.

In our preliminary studies, we screened the effects of antibacterial activities of 10 ITCs on *S. aureus*, and found MTPITC, PEITC, and BITC had relatively high capacities. Therefore, we selected the three ITCs as the main subjects in the present study. γ-CD and ITCs were mixed at a mass ratio of 1:1, and γ-CD-ITC inclusion complexes were obtained by freeze-drying. UV–visible spectroscopy is an important tool to study the complexation of ITCs with γ-CD. As shown in Figure S1, the UV absorption spectra were recorded for γ-CD and the inclusion complexes. In comparison with γ-CD, the inclusion complexes had a maximal absorption peak at 247 nm, which was attributed to the N=C=S group in ITCs. However, the formation of ITCs inclusion complexes with γ-CD remained uncertain. Figure 2a showed the FTIR spectra of free ITCs, γ-CD, and γ-CD-ITCs. The characteristic broad peak in the FTIR spectra of ITCs at 2140–2040 cm^−1^ was attributed to the N=C=S group [14]. The spectrum of γ-CD showed notable peaks ranging from 3540 cm^−1^ to 3230 cm^−1^ due to hydroxyl group stretching vibrations [34]. The main absorption bands were observed at 2926 cm^−1^ (C–H vibration) and 1027 cm^−1^ [34] for the C–O group vibration. Although characteristic benzene peaks (between 1345 cm^−1^ and 1600 cm^−1^) [34] vanished in the spectra of γ-CD-PEITC and γ-CD-BITC, the vibration bands of N=C=S groups still existed. The γ-CD-MTPITC, γ-CD-BITC, and γ-CD-PEITC peaks were not significantly different. These results indicated that the ITCs were successfully encapsulated by γ-CD. These findings were consistent with previously reported studies on the inclusions of ITCs with β-CD, in which only the peaks corresponding to N=C=S groups were detected in the ITC inclusion complexes, which correctly considered that the structure in ITCs was completely wrapped except for the N=C=S group [34,35].

TGA was used to evaluate the association between the temperature change and weight loss of the complexes, providing data on the thermal stability and starting composition. ITCs are volatile compounds with a mass loss at temperatures ranging from 80 °C to 165 °C [15,34]. As shown in Figure 2b, γ-CD and γ-CD -ITC inclusion complexes decomposed in two steps. The first procedure was to dehydrate the γ-CD sample from 30 °C to 132 °C. The TGA spectra of γ-CD-MTPITC, γ-CD-PEITC, and γ-CD-BITC were compared to γ-CD as the standard host moiety, and the decomposition tracks of the three γ-CD-ITCs were very similar. In fact, due to the effect of γ-CD, the phased mass loss of the γ-CD-ITC inclusion complexes showed an evident slope between 132 °C and 300 °C, which delayed their volatilization. A similar study of the inclusion complex of efavirenz with γ-CD was reported by Braga et al. [36]. In addition, both BITC and carvacrol inclusion complexes encapsulated in β-CD have high thermal stabilities [34,37]. Therefore, the elevated temperature required for the complete decomposition of ITCs further confirmed the formation of ITC inclusion complexes with γ-CD.

XRD is a useful tool for determining CD complexes in powder or microcrystalline forms [38]. Due to ITCs being liquid with high volatility, the crystal structure of ITCs is difficult to detect. XRD diffraction patterns of γ-CD, γ-CD-MTPITC, γ-CD-PEITC, and γ-CD-BITC complexes were further investigated. As shown in Figure 2c, γ-CD exhibited solid and sharp peaks at 2θ values of 12.42°, 16.44°, 18.62°, 20.36°, and 23.32°, which confirmed the crystalline nature of γ-CD. The sharp peaks at 2θ of 5.84–5.90°, 10.28–10.32°, 11.80–11.84°, 15.74–15.90°, and 21.80–21.86° were similar in the three γ-CD-ITCs. Sharp peaks at 14.21° appeared in the XRD patterns of γ-CD-BITC and γ-CD-PEITC, but not γ-CD-MTPITC, which may represent a crystal morphology unique to the inclusion complexes containing a benzene ring structure [35]. In comparison, the XRD spectra of γ-CD-ITCs were clearly distinct from that of γ-CD. Similar results were obtained for BITC-β-CD, showing a crystal form different from that of β-CD [34]. Therefore, the differences in the structures and thermal stability of γ-CD and γ-CD-ITCs further confirmed the formation of inclusion complexes.

### 3.2. Antibacterial Activities of ITCs and γ-CD-ITCs against S. aureus

**Figure 3:**
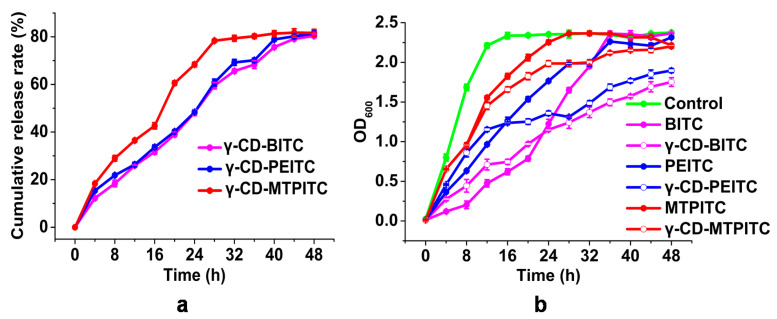
(**a**) In vitro release behaviors of ITCs from γ-CD-ITCs at 37 °C. (**b**) Growth curves of *S. aureus* treated with the control, ITC (0.25 mmol/L), and γ-CD-ITC (0.25 mmol/L ITC). Values are means ± standard deviations, indicated by error bars.

The curves for in vitro release of ITCs from γ-CD-ITCs at 37 °C are shown in Figure 3a. The cumulative release of γ-CD-BITC, γ-CD-PEITC, and γ-CD-MTPITC was increased with the incubation time, and reached to 81.4, and 81.7% at 48 h, representing a significant burst effect. Figure 3b showed the effects of ITCs and γ-CD-ITCs on the growth curve of *S. aureus*. The order of antibacterial activity of these three ITCs against *S. aureus* was BITC>PEITC>MTPITC. The antibacterial effect of inclusion complex was exceeded to the corresponding ITCs after 24, 20, and 12 h for γ-CD-BITC, γ-CD-PEITC, and γ-CD-MTPITC, respectively. The best controlled release effect was found in γ-CD-BITC. Moreover, the results also indicated that the release efficiency of the inclusion complexes correlated significantly with the antibacterial activity with Pearson correlation coefficient from 0.942 to 0.994. As shown in Table 1, the antibacterial activities of the different ITCs and γ-CD-ITCs were preliminarily evaluated using the agar diffusion assay. On the first day, the inhibition zone sizes of the three ITCs against *S. aureus* were 7.9 ± 0.4, 10.8 ± 0.4, and 16.9 ± 0.7 mm, respectively, following the order of BITC>PEITC>MTPITC. Consistent with previous studies, BITC showed greater antibacterial activity than PEITC against Shiga toxin-producing *Escherichia coli* and enterotoxigenic *E. coli* [13]. No significant differences in the size of the inhibition zone were observed between the three ITCs and their corresponding inclusion complexes on the first day (*p* > 0.05). Neither MTPITC nor PEITC exhibited antibacterial activities by the third day, while their inclusion complexes still displayed antibacterial activities, and the antibacterial effect of γ-CD-PEITC lasted up to 5 days. The antibacterial activity of BITC persisted until the third day. Due to the volatilization of ITCs, the antibacterial activities decreased with time. After encapsulation of γ-CD, the antibacterial activity of γ-CD-BITC was still stronger on Day 10 with an inhibition zone of 7.2 ± 0.9 mm. Although the antibacterial activities still showed a gradual decrease over time for the γ-CD-ITC inclusion complexes, the controlled release ability was significantly improved after encapsulation compared to that of free ITCs. Furthermore, BITC had an MIC of 0.5 mmol/L against *S. aureus* in this study. Overall, BITC was the most active ITC, consistent with results from previous study that evaluated 10 ITCs and found that BITC exerted the greatest inhibitory effects on foodborne pathogens [10]. It has been reported that a significant synergic effect was evidenced when norfloxacin was combined with the ethyl and propyl esterified derivatives, suggesting that lipophilicity plays an important role in the antibacterial activity [39]. However, BITC is a highly volatile substance limiting its wide application [40]. Uppal et al. showed that the antibacterial activities of BITC chitosan nanoparticles (preparations) were greater than those of BITC by performing an inhibition zone assay and determining the MIC [15]. Moradi et al. reported that thyme oil inclusion complexes with γ-CD showed a more controlled release than hydroxypropyl-β-CD and methyl-β-CD [26]. It was observed that the inclusion complex α-bisabolol/β-CD demonstrated a direct antibacterial effect upon *S. aureus*, in combination with gentamicin [23]. In view of the good antibacterial ability of BITC and the good sustained release of its inclusion complex with γ-CD, we selected BITC and γ-CD-BITC as the main subjects in the following studies.

### 3.3. Effects of BITC and γ-CD-BITC against S. aureus Biofilm Formation

**Figure 4:**
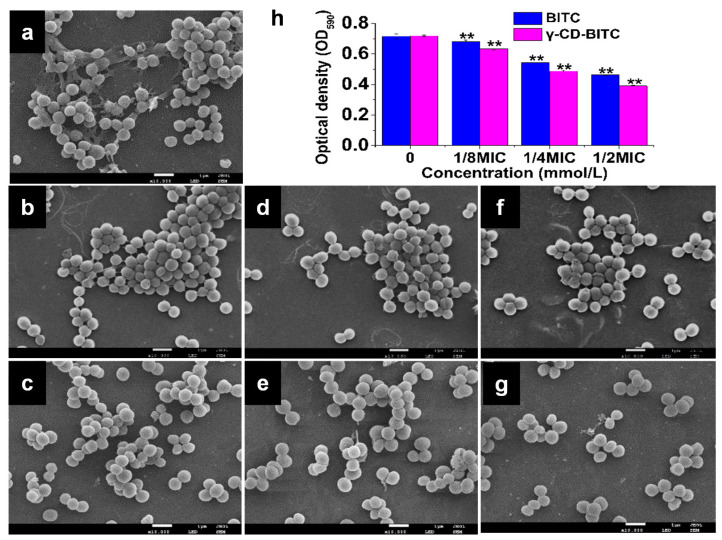
Effects of BITC and γ-CD-BITC on *S. aureus* biofilm formation. SEM images (×10,000) of *S. aureus* biofilms (**a**), control; (**b**), 1/8 MIC BITC; (**d**), 1/4 MIC BITC; (**f**), 1/2 MIC BITC; (**c**), 1/8 MIC γ-CD-BITC; (**e**), 1/4 MIC γ-CD-BITC; (**g**), 1/2 MIC γ-CD-BITC). (**h**) Crystal violet assay. Each bar represents the mean ± SD of three independent experiments, ** *p* < 0.01 compared with the control group.

The inhibitory effects of BITC and γ-CD-BITC at 0–1/2 MIC (basing on the BITC concentration) on biofilm formation by *S. aureus* were investigated. As shown in Figure 4a–g, the inhibitory effects of BITC and γ-CD-BITC on the development of the *S. aureus* biofilms were observed using SEM. The untreated *S. aureus* showed that the biofilm was highly dense, with numerous bacteria wrapped in the biofilm structure (Figure 4a). In contrast, the biofilms were thinner in both the BITC and γ-CD-BITC groups, and fewer bacteria were enveloped as the BITC concentration increased (Figure 4b–g). More importantly, both biofilms and bacteria in the γ-CD-BITC group were reduced, compared to the group treated with BITC at the same concentration (Figure 4d,f). Furthermore, as shown in Figure 4h, biofilm formation by *S. aureus* decreased as the BITC concentration increased, as determined using the crystal violet quantitative assay. Significantly less biofilm formation by *S. aureus* treated with γ-CD-BITC was observed than bacteria treated with BITC at each concentration. This phenomenon is due to the high volatility of BITC, while the controlled release of the γ-CD-BITC inclusion complex significantly attenuates this weakness. Free anacardic acid (AnAc) reduced *S. aureus* biofilms by 92.1% at 50 μg/mL for 72 h, while the AnAc inclusion complex with hydroxypropyl-β-CD (AnAc-HP-β-CD) displayed full biofilm dispersion at 25 μg/mL [41]. In addition, AnAc-HP-β-CD showed a better ability to eradicate *S. aureus* biofilms than free AnAc [41]. The biofilm-forming ability of *S. aureus* treated with γ-CD/usnic acid films was impaired and the antibacterial properties of the γ-CD/ usnic acid films were maintained for at least 72 h compared to the control, achieving long-term controlled release [42]. Therefore, BITC encapsulated in γ-CD is more effective than free BITC at inhibiting *S. aureus* biofilm activities.

### 3.4. Effects of BITC and γ-CD-BITC on Virulence Gene Expression in S. aureus

**Figure 5:**
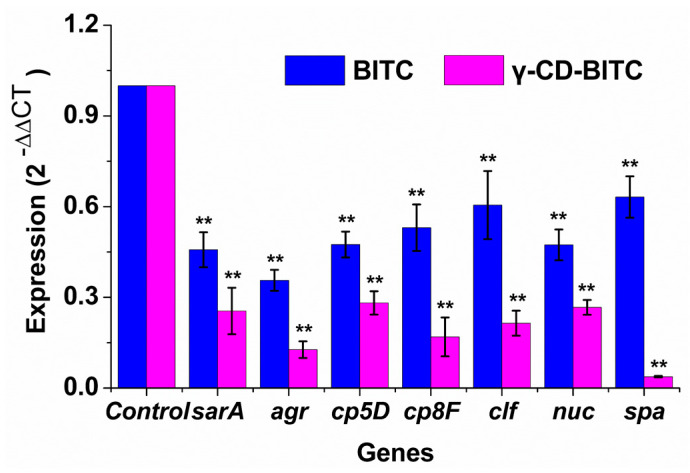
Effects of BITC and γ-CD-BITC on virulence gene expression in *S. aureus*. Relative expression of *sarA*, *agr*, *cp5D*, *cp8F*, *clf*, *nuc*, and *spa* compared to 16S rRNA after normalization to one control. The data were calculated using the average of three independent replicates. ** Differences in gene expression were significant (*p* < 0.01).

RNA was extracted from *S. aureus* treated with or without 1/4 MIC BITC and γ-CD-BITC for 24 h. The effects of γ-CD-BITC and BITC on the expression of virulence genes in *S. aureus* was then observed using qRT-PCR. As shown in Figure 5, γ-CD-BITC showed stronger inhibitory effects on these virulence genes in *S. aureus* than BITC. The relative expression of the *sarA*, *agr*, *cp5D*, *cp8F*, *clf*, *nuc*, and *spa* genes was 12.7–37.4% and 35.6–63.2%, respectively, in groups treated with γ-CD-BITC and BITC. Therefore, due to the controlled release effect of the γ-CD-BITC inclusion complex, it is more effective than free BITC at inhibiting the expression of virulence genes.

Toxin generation and biofilm development are two well-known contributors to *S. aureus* infection. The spa gene encodes staphylococcal protein A, an essential *S. aureus* pathogenicity factor required for the adherence and aggregation of bacteria during the development of biofilms [43]. γ-CD-BITC reduced the expression of the *spa*. Therefore, we hypothesize that γ-CD-BITC reduces the formation of biofilms by reducing *S. aureus* adhesion and aggregation. In addition, Liu and Liang et al. also found that the downregulation of *spa* and *sarA* reduced biofilm formation by *S. aureus* [44,45]. Quorum sensing by the accessory gene regulatory system is linked to staphylococcal pathogenicity [46]. Furthermore, accumulating data suggests that the *agr* phenotype is required for a variety of biofilm characteristics, including the capacity to adhere to surfaces and disperse, and may even influence the duration of biofilm-associated illnesses [47,48,49]. *S. aureus* evades ingestion and death through *CP5* and *CP8*, which are major virulence factors [50,51,52]. Thermonuclease (*nuc*) is a specific virulence factor of *S. aureus* that is routinely used to identify samples [53,54]. Furthermore, the adhesion gene aggregation factor (*clf*) is closely linked to biofilm development, and its protein facilitates fibrinogen adhesion [55].

### 3.5. Primary Model for the Inhibitory of BITC and γ-CD-BITC on S. aureus Growth in Cooked Chicken Breasts

**Figure 6:**
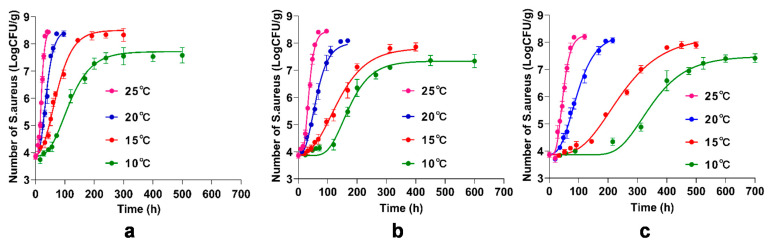
The Primary model for the inhibitory of BITC and γ-CD-BITC on *S. aureus* growth in cooked chicken breast stored at 10, 15, 20, and 25 °C. (**a**) Control, (**b**) BITC, and (**c**) γ-CD-BITC.

The primary models are developed using the modified Gompertz model, which is applied to a number of predictive models with modified equations. It has been adapted to assess the growth of *S. aureus* in animal foods such as raw beef, beef jerky, and smoked chicken [32,56,57]. In this study, chicken breasts were inoculated with *S. aureus*, treated with BITC and γ-CD-BITC, and stored at 10, 15, 20, and 25 °C. Figure 6 presented the growth of *S. aureus* in cooked chicken breasts treated with or without BITC and γ-CD-BITC. The R^2^ values of the modified Gompertz models were 0.9783–0.9863, 0.9816–0.9915, and 0.9816–0.9916 for the control, BITC, and γ-CD-BITC models at 10, 15, 20, and 25 °C, respectively. Bacterial growth on chicken breasts at different storage temperatures showed a similar pattern to that of *S. aureus* in pork [57]. Therefore, the modified Gompertz model accurately predicted the growth of *S. aureus* in cooked chicken breasts treated with and without BITC and γ-CD-BITC.

### 3.6. Secondary Model for Growth Factors of the Inhibitory of BITC and γ-CD-BITC on S. aureus in Cooked Chicken Breasts

**Figure 7:**
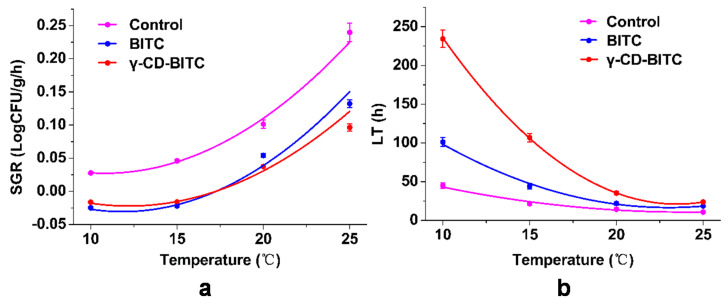
The secondary model for growth factors of the inhibitory effects of BITC and γ-CD-BITC on *S. aureus* in cooked chicken breast stored at 10, 15, 20, and 25 °C. (**a**) SGR, maximum specific growth rate and (**b**) LT, lag time.

According to Yu et al. [32], the secondary model equation was created using the modified equations of the Gompertz model to get the indices, including lag time (LT) and maximum specific growth rate (SGR). Table S2 presents secondary model growth parameters for predicting *S. aureus* fate after treatment with BITC and γ-CD-BITC. A polynomial equation was used to characterize the effects of temperature and antibacterial agents on *S. aureus* growth. Figure 7 shows the secondary models for growth factors of *S. aureus* inoculated in cooked chicken breast and treated with BITC and γ-CD-BITC at 10, 15, 20, and 25 °C. The change in the SGR of *S. aureus* in cooked chicken breast samples according to temperature and antibacterial substances was presented in Figure 7a. The SGR of samples treated with both BITC and γ-CD-BITC was significantly decreased compared to that of the control at each temperature. However, no significant difference in the SGR of *S. aureus* was observed at 10 and 15 °C in samples treated with BITC and γ-CD-BITC, but samples treated with γ-CD-BITC had a significantly lower SGR than samples treated with BITC at 20 and 25 °C. In addition, as shown in Figure 7b, the temperature and antibacterial substances changed the LT of *S. aureus* in cooked chicken breasts. The LT showed a tendency to decrease as the experimental temperature increased upon treatment with or without BITC and γ-CD-BITC. The LT of samples treated with γ-CD-BITC was 1.3–2.4 times longer than that of BITC at 10, 15, 20, and 25 °C. This phenomenon was due to the high volatility of BITC [15], while the controlled release of γ-CD-BITC prolonged the LT. Therefore, γ-CD-BITC and a low temperature are required to increase the LT of *S. aureus* in cooked chicken breasts.

Table S3 showed the validation of the *S. aureus* growth factors in the secondary model. The Bf, Af, and R^2^ of the secondary models were 0.94–1.02, 1.00–1.23, and 0.90–0.99, respectively. The relative deviation between the observed and anticipated data is denoted by Bf. The model is deemed inappropriate if the computed value exceeds the range of 0.7–1.5 [33,58]. Af is the value of the absolute difference between the actual value and anticipated value of a secondary model parameter. The closer the computed number is to 1, the more exact the model [59]. Bf and Af for the secondary model were 1.014–1.188 and 1.014–1.190, respectively, in LT and SGR, in the prediction model of *S. aureus* in pork [57]. For the description of growth factors in *S. aureus* in samples treated with BITC and γ-CD-BITC compared with the findings of other studies, the validation results for the secondary model were acceptable. Therefore, γ-CD-BITC also demonstrated a controlled release effect against *S. aureus* in chicken systems.

## 4. Conclusions

The present study showed the successful embedding of BITC in γ-CD, overcoming the lacunas of the high volatility of BITC. Compared with BITC, γ-CD-BITC showed more sustained inhibition of *S. aureus*. Both biofilm formation and the expression of virulence genes were lower in *S. aureus* treated with γ-CD-BITC than in bacteria treated with BITC. In addition, this study more accurately predicted the growth tendencies of *S. aureus* based on temperature, time, and antibacterial substances using a modified Gompertz model and polynomial model equation. These results suggested that γ-CD was a suitable excipient for increasing the stability of BITC, and the γ-CD-BITC inclusion complex improved controlled release to inhibit *S. aureus*. This work provided the theoretical information for the characterization of γ-CD-BITC that could be potentially utilized as a good substance in the preservation of chicken foods.

## Figures and Tables

**Table 1:** The diameters of inhibition zone of ITCs and γ-CD-ITCs against *S. aureus*.

Times (Day)	Inhibition Zone (mm)						
	Control	MTPITC	PEITC	BITC	γ-CD-MTPITC	γ-CD-PEITC	γ-CD-BITC
1	0.00 ± 0.00 ^Aa^	7.9 ± 0.4 ^Bb^	10.8 ± 0.4 ^Bc^	14.9 ± 0.7 ^Cd^	8.1 ± 0.5 ^Cb^	10.8 ± 0.6 ^Dc^	14.7 ± 0.6 ^Dd^
3	0.00 ± 0.00 ^Aa^	0.00 ± 0.00 ^Aa^	0.00 ± 0.00 ^Aa^	9.1 ± 0.25 ^Bc^	3.1 ± 0.42 ^Bb^	9.1 ± 0.6 ^Cc^	11.1 ± 0.7 ^Cd^
5	0.00 ± 0.00 ^Aa^	0.00 ± 0.00 ^Aa^	0.00 ± 0.00 ^Aa^	0.00 ± 0.00 ^Aa^	0.00 ± 0.00 ^Aa^	6.3 ± 0.6 ^Ba^	9.1 ± 0.9 ^Bb^
10	0.00 ± 0.00 ^Aa^	0.00 ± 0.00 ^Aa^	0.00 ± 0.00 ^Aa^	0.00 ± 0.00 ^Aa^	0.00 ± 0.00 ^Aa^	0.00 ± 0.00 ^Aa^	7.2 ± 0.9 ^Ab^

Zones of growth inhibition values are represented as mean ± standard deviation. Means with different superscripts a–c in a row and A−B in a column differ significantly (*p* < 0.05).

## Data Availability

The data that support the findings of this study are available within the article.

## Supplementary Materials

The following are available online at https://www.mdpi.com/article/10.3390/foods11010060/s1, Figure S1: UV–vis absorption spectrum of γ-CD and γ-CD-ITCs, Table S1: Sequences of primers used in qRT-PCR, Table S2: Growth parameters of secondary models to predict the growth of *S. aureus* under BITC and γ-CD-BITC, Table S3: Development and validation of secondary models for growth factors of *S. aureus* in cooked chicken breast under BITC and γ-CD-BITC.
