# Supplementary material for: Characterization of the Inclusion Complexes of Isothiocyanates with γ-Cyclodextrin for Improvement of Antibacterial Activities against Staphylococcus aureus
Source: Foods. 2021 Dec 27;11(1):60. doi: 10.3390/foods11010060 (PMC8750663; doi:10.3390/foods11010060)
Supplement: Supplementary file 1 [file foods-11-00060-s001.zip › foods-1489555-supplementary.pdf]

Figure S1. UV–vis absorption spectrum of γ-CD and γ-CD-ITCs

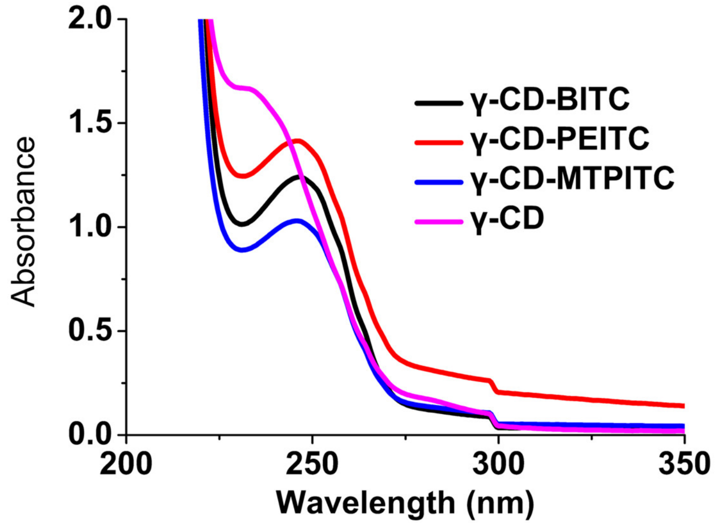

Table S1. Sequences of primers used in qRT-PCR

Gene	Primer	Sequence (5′–3′)
16S rRNA	*16S rRNA-F*	CGTGCTACAATGGACAATACA
	16S rRNA-R	ACAATCCGAACTGAGAACAAC
Cp8F	*Cp8F-F*	ACAGACTTTAGTTATCCCTTAC
	Cp8F-R	TGATGCCAGTGATTACCTTTA
CP5D	*CP5D-F*	CTTTAGTTGTTGGTGCTGGTC
	CP5D-R	CGGTTCAAGTTTCATTTCGTC
nuc	*nuc-F*	GAAAGGGCAATACGCAAAG
	nuc-R	ACGCCATTATCTGTTTG
spa	*spa-F*	ATAAGAAGCAACCAGCAAAC
	spa-R	GGCTAATGATAATCCACCAA
clf	*clf-F*	ACGAATGGCGATGTTGTAGC
	clf-R	CTCGGTCTGTAAATAAAGGTAATG
SarA	*SarA-F*	ATGATTGCTTTGAGTTGTTA
	SarA-R	TTTGTTTTCGCTGATGTATG
agr	*agr-F*	AGACCTGCATCCCTAATCGTA
	agr-R	TCAGTTTGCCACGTATCTTCA

Table S2. Growth parameters of secondary models to predict the growth of *S. aureus* under BITC and γ-CD-BITC

Parameter	Temperature (°C)	Sulfur-containing spices		
		**Control**	**BITC**	**γ-CD-BITC**
SGR	10	0.03±0.00^{Aa}	-0.02±0.00^{Ab}	-0.02±0.00^{Ab}
	15	0.05±0.00^{Aa}	-0.02±0.00^{Ab}	-0.02±0.00^{Ab}
	20	0.10±0.01^{Ba}	0.05±0.00^{Bb}	0.04±0.00^{Bc}
	25	0.24±0.01^{Ca}	0.13±0.01^{Cb}	0.10±0.01^{Cb}
LT	10	45.09±3.6^{Aa}	101.20±6.1^{Ab}	234.30±11.17^{Ac}
	15	21.77±2.3^{Ba}	44.34±3.7^{Bb}	106.90±5.37^{Bc}
	20	14.39±1.4B^{Ca}	22.10±2.2^{Ca}	35.34±2.2^{Cb}
	25	10.90±0.60^{Ca}	18.51±0.81^{Cb}	23.89±1.3^{Cc}

SGR, maximum specific growth rate; LT, lag time; T, temperature. $P<0.05$.

Table S3. Development and validation of secondary models for growth factors of *S. aureus* in cooked chicken breast under BITC and γ-CD-BITC

Sulfur-containing spices	Secondary model equations	Bf	Af	R^2
Control	SGR = 0.14211-0.02123×T + 0.0009819 × T^2	1.00	1.04	0.97
	LT = 105.60–7.892×T + 0.16423 × T^2	1.00	1.08	0.94
BITC	SGR = 0.11667-0.02484×T + 0.00105 × T^2	0.94	1.23	0.90
	LT = 272.22–22.22×T + 0.48278 × T^2	1.00	1.05	0.98
γ-CD-BITC	SGR = 0.10784-0.02128×T + 0.0008718 × T^2	1.02	1.21	0.91
	LT = 671.37–55.37×T + 1.1789×T^2	1.00	1.00	0.99

SGR, maximum specific growth rate; LT, lag time; T, temperature. Bf, bias factor; Af, accuracy factor; R^2, correlation coefficient.
